# Malignant Neglect: The Failure to Address the Need to Prevent Premature Non-communicable Disease Morbidity and Mortality

**DOI:** 10.1371/journal.pmed.1001466

**Published:** 2013-06-11

**Authors:** David Stuckler, Sanjay Basu

**Affiliations:** 1 Department of Sociology, University of Oxford, Oxford, United Kingdom; 2 Prevention Research Center, Stanford University, Stanford, California, United States of America

## Abstract

David Stuckler and Sanjay Basu comment on a study by Carl Lachat and colleagues documenting the lack of policies addressing noncommunicable disease prevention in low- and middle-income countries and outline steps for making such policies accessible, effective, and transparent.

**Linked Research Article**

This Perspective discusses the following new study published in *PLOS Medicine*:

Lachat C, Otchere S, Roberfroid D, Abdulai A, Seret FMA, et al. (2012) Diet and Physical Activity for the Prevention of Non Communicable Diseases in Low and Middle-Income Countries: A Systematic Policy Review. PLoS Med 10(6): e1001465. doi:10.1371/journal.pmed.1001465

Watching a slow-motion disaster unfold is a vexing public-health challenge. It is particularly frustrating when policymakers, aware of the problem, fail to respond. The histories of climate change, HIV, and famine are prominent examples of such malignant neglect—that is, “doing harm by doing nothing.”

The global burden of non-communicable disease (NCD) would, on first brush, not seem to suffer from a lack of policy activity. Recent high-level policy discussions have targeted NCDs. In 2000, ministers from 193 World Health Organization (WHO) member states adopted a Global Strategy for the prevention and control of NCDs at the World Health Assembly [1]. Subsequently, in 2001, the WHO surveyed over 130 member states about their awareness of and response to the high and rising burden of NCDs [2]. Then in 2004, WHO member states affirmed their commitment to addressing diet and physical activity through cost-effective interventions [3],[4]. About one decade later, the United Nations held a High Level Meeting on NCDs at the UN General Assembly, only the second time such a high level meeting occurred for a public health threat (the previous one being on HIV, in 2001) [5]. Over 180 leaders of member states again agreed to implement cost-effective measures to curb the avoidable burden of premature deaths from NCDs, and to address the social and economic consequences of these diseases for households [6]. However, despite these voluntary declarations to act on the avoidable burden of NCDs, the systematic review by Carl Lachat and colleagues in this week's issue of *PLOS Medicine* reveals that disappointingly little has changed: despite some policy statements, there remains virtually no development of specific policies and programs to address NCDs in the countries facing the highest rates of premature morbidity and mortality. Lachat and colleagues performed a structured analysis of health policies and programs to address diet and physical activity risk factors for NCDs in 116 of the 140 low- and middle-income countries that are WHO members [7]. The authors found that health policies now exist on the books, *de jure*, in 72% of these countries. However, within the 116 countries NCD prevention programs that address intake of salt, fat, fruit, and vegetable or the promotion of physical activity were found in only 47% (54 countries). A minority of countries have implemented some of the most effective regulatory actions [8],[9]) to address unhealthy diets or measures to encourage physical activity—two risk factors that account for about 40% of the avoidable burden of NCDs. Of these NCD programs, few include a budget, implementation plan, time frame, or statement of which government entity is responsible for making progress toward the declared goal. The majority of programs focus on increasing consumers' awareness of health risks through abstract informational campaigns. There were no policy actions found for counteracting vested interests, including ultra-processed food, alcohol, and tobacco companies, which are driving consumer trends at present. Hence, it was not possible to ascertain whether and the extent to which *de jure* policies are being met with action on the ground, *de facto*.

## Reasons for Malignant Neglect of NCDs

Two key features of the study underscore some reasons for the malignant neglect of NCDs. First, there is virtually no serious response to potential conflicts of interest with industries that manufacture and market the products directly attributable for rising NCDs, as covered in *PLOS Medicine*'s Big Food series (http://www.ploscollections.org/article/browse/issue/info%3Adoi%2F10.1371%2Fissue.pcol.v07.i17 ) [10]. Left unaddressed, manufacturers of unhealthy commodities will continue to spread their products, so long as profit margins remain positive.

Second, there is a vast divide between resources available to the public and those used to construct policy. The researchers undertook a systematic, transparent process to identify the availability of policies on NCDs. Yet, despite this rigorous and intensive process, the World Health Organization Nutrition, Obesity, and Physical Activity Database (NOPA, http://data.euro.who.int/nopa/), a collaborative project of the European Commission and WHO, did not include access to the actual policies in all cases, and language limitations precluded analysis of documents for Azerbaijan, Belarus, and Russia, among other countries.

## Holding Politicians Accountable

How can policymakers be held to account for their actions (or inactions) without accessible data on policies and their implementation? As one concrete example, little is known about why Russia, Moldova, and Czech Republic have achieved substantial reductions in age-standardized NCD mortality rates since 2005 [11]. Without policy data, it remains speculative as to what could have been the underlying reason for their latest successes. Was it a result of intentional NCD policies? An artifact of changes in data collection or surveillance? An unintentional result of market changes affecting the affordability of ultra-processed foods, tobacco, and alcohol? A natural shift of population behavior with changing socio-economic context? Only with accurate, systematically collected and longitudinal data on *both* NCD policies and outcomes will it be possible for researchers to hold policymakers (and the vested interests whom they sometimes serve) accountable for their success and failure to curb rising NCD burdens [12].

A clear next step is to extend global monitoring systems, such as the WHO Global Monitoring Framework, to cover NCDs and the policies that aim to reduce them. The World Health Assembly's resolution in June 2013 for the first time to monitor nine global NCD targets and 25 NCD indicators for all WHO members is a critical starting point. Resources are lacking, however. The privately organized Global Burden of Disease Project, with the support of the Bill & Melinda Gates Foundation, has vastly helped to identify the increase in NCDs. Yet many statistics from that project are “imputed” estimates, meaning that little or no on-the-ground data are available from many countries to tell us what is happening to people as they are increasingly exposed to NCD risks. The next generation of data philanthropy should rise to the challenge of understanding policy success and failure. People around the world deserve to know which policies they are (or are not) being exposed to and what impact these policies have on them.

## Abbreviations

NCD: non-communicable disease
WHO: World Health Organization
